# Supplementary figures and images for: Ginseng Sprouts Attenuate Mortality and Systemic Inflammation by Modulating TLR4/NF-κB Signaling in an LPS-Induced Mouse Model of Sepsis
Source: Int J Mol Sci. 2023 Jan 13;24(2):1583. doi: 10.3390/ijms24021583 (PMC9860726; doi:10.3390/ijms24021583)

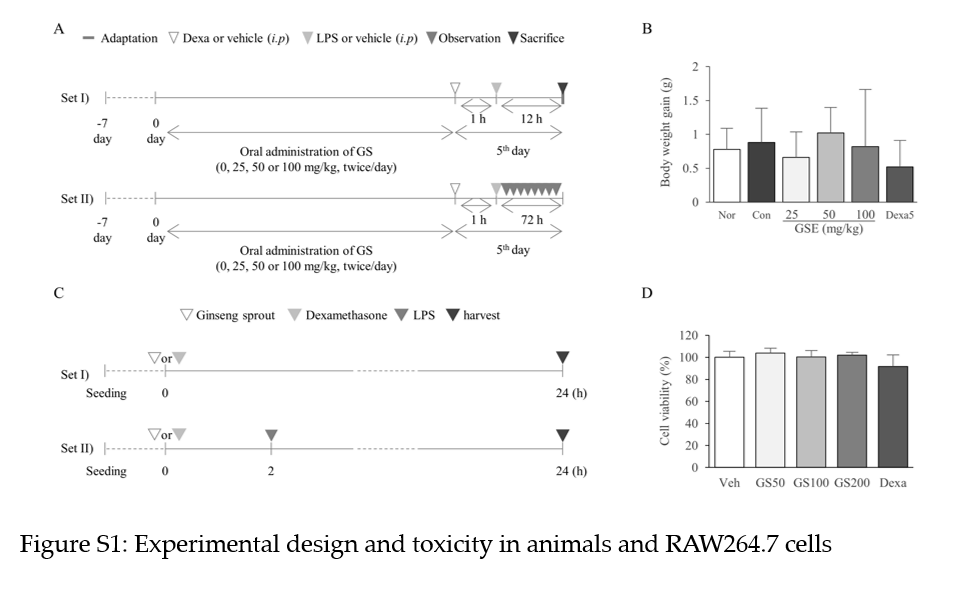

Supplement: Supplementary file 1 [file ijms-24-01583-s001.zip › Figure S1.png]

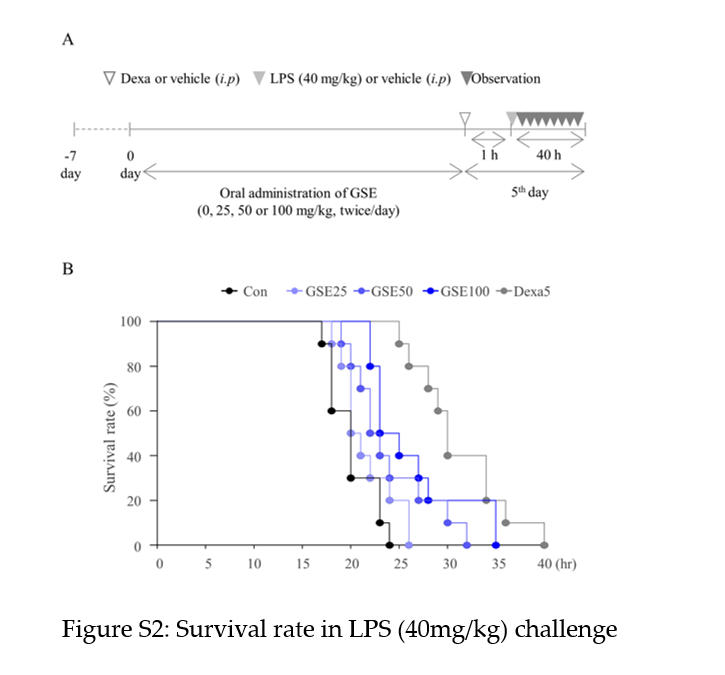

Supplement: Supplementary file 1 [file ijms-24-01583-s001.zip › Figure S2.png]

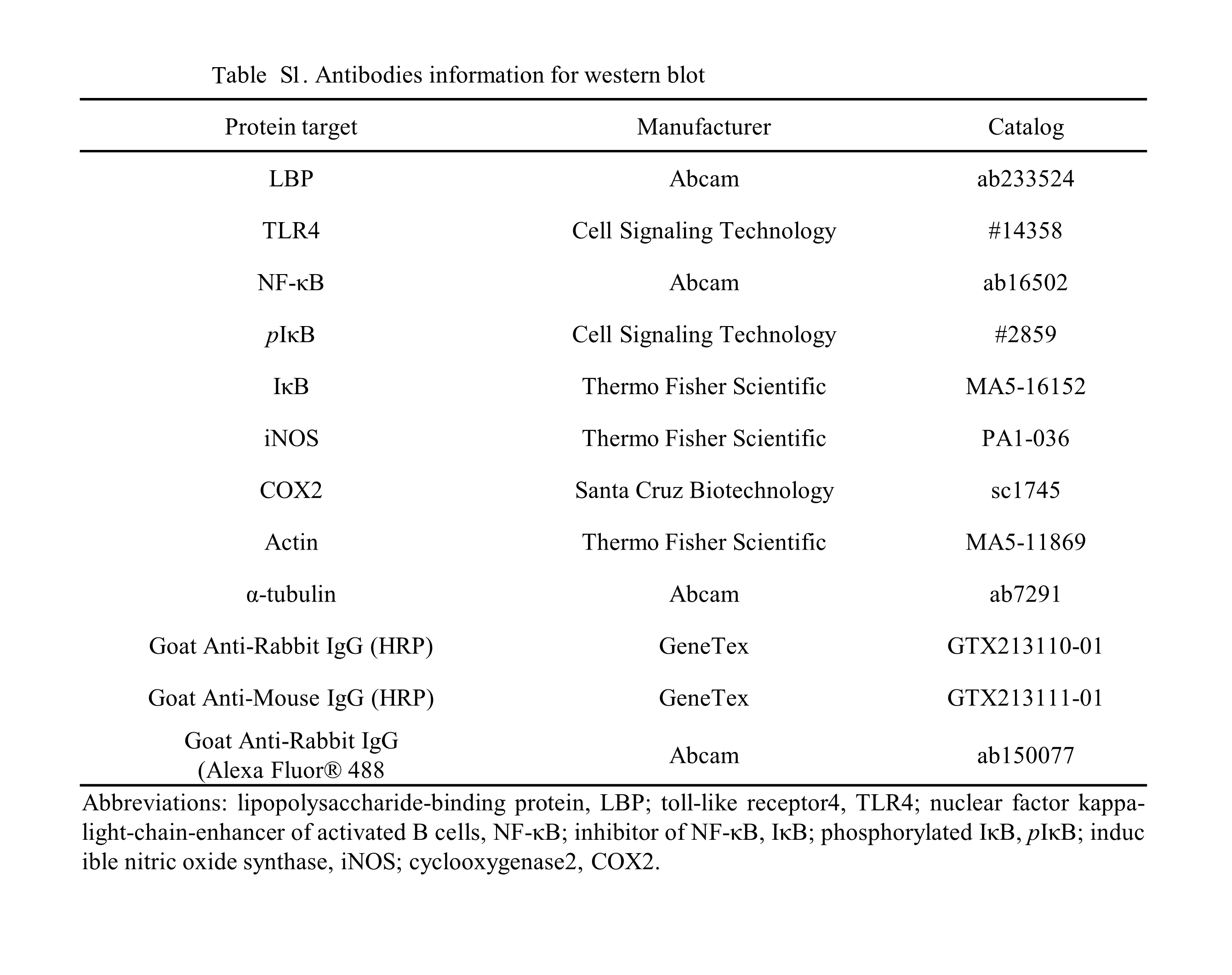

Supplement: Supplementary file 1 [file ijms-24-01583-s001.zip › Table S1.png]
